# Lectures on Practical and Medical Surgery, &c.

**Published:** 1830-07-01

**Authors:** 


LECTURES ON PRACTICAL AND MEDICAL SURGERY, COMPRISING OBSERVATIONS AND REFLECTIONS ON SURGICAL EDUCATION; ON THE INVESTIGATION OF DISEASE; AND ON THE ORDINARY DUTIES OF THE SURGEON: FORMING PART OF AN EXTENDED COURSE ON THE PRINCIPLES AND PRACTICE OF SURGERY, DELIVERED IN 1828 AND 1829. Illustrated by Engravings. By *Thomas Alcock.* Member of the Royal College of Surgeons, &c. &c. With coloured Plates, and with uncoloured; pp. 302. London, 1830.

IN a former number of this Journal we had an opportunity of speaking favourably of the talented and observant author of these Lectures, while reviewing his Essay on Medical Education in the Transactions of the Associated Apothecaries. It is true, we differed from Mr. Alcock on some points connected with the apprenticeship system, and if we may judge by the following passage, Mr. Alcock drew a hornet's nest about his ears by the admonitions addressed to the masters or preceptors of apprentices.

"I soon found, that the fate of the *Miller, his Son, and their Ass,* in the fable, awaited my opinions on this subject.

"Some of my young friends who were apprentices, and not a few of others in practice, who had tasted of the inconveniencies arising from ill assorted engagements of this kind, wondered that I did not explode apprenticeships altogether.

"The greater part of masters (for the term preceptors did not suit them,) accosted me, 'A pretty piece of work you have cut out for us! Who do you think can submit to the drudgery of giving daily instruction to his apprentice? The idea is absurd!'

"Another class, and they were for the most part parents themselves, who had experienced the anxieties, the arduous responsibility of duties for which no one can be too well prepared, greeted my efforts with their cordial approbation, and added, that they felt assured, had such a system of instruction been pursued in their own education, it would have saved them much valuable time, and have obviated many of the anxieties which they had suffered." 14.

In another part of the same lecture from which we have taken the above extract, Mr. Alcock acknowledges that a general practitioner, who is in any thing like good practice, has not time to study himself, or keep on a level with the progress of the literature, art, and science of the day. How then is he to pay all the attention which Mr. A. recommends to the studies of his pupils? We do not say that he ought not to give this minute attention to his apprentices. God forbid! We know that it is his duty, as a Christian, to prefer the good of others to his own, and therefore even at the sacrifice of emolument as well as time, he *should* do as Mr. Alcock desires. What *is*, and what *ought to be*, are very different things. "Love thy neighbour as thyself," is a precept in the mouth of every one—but practised by nobody! We need not, however, dwell on this subject at present. This volume of lectures, although modestly intended for those who have not yet acquired experience, will be found to contain a series of most useful practical observations and instructions on the minor, but more common operations and duties of the medical practitioner, and more especially the general practitioner. The elementary nature of the work, and the great minuteness with which Mr. Alcock treats the details of medical and surgical practice—the investigation of disease—and the discrimination of its varying characters—render it quite impossible to attempt an analysis. The only portion of the volume which will bear this process is a section entitled :—

"Observations on the Inflammations of the Mucous Membranes of the Organs of Respiration, and to this we shall direct our attention.

Our author is convinced that the strict relations existing between these inflammations and some other diseases, especially measles, scarlatina, variola, and pertussis, have not been sufficiently considered by practitioners or pathologists. The following is the arrangement which Mr. Alcock proposes, founded on the situation of the parts affected.

"A. The nostrils, the fauces, and the mouth.
"B. The larynx; the top of the pharynx; as the latter is generally implicated in inflammation of the larynx.
"C. The trachea.
"D. The bronchia.
"To the above division A. appertain some forms of Catarrh, Aphtha.
"Cynanche tonsillaris.
"——— maligna.
"To B. Cynanche laryngea, or laryngitis; Cynanche Pharyngea.
"To C. Cynanche trachealis, or croup.
"To D. Bronchitis.

"To the second order B, (sometimes including the third and fourth, C and D,) chiefly appertain those accidental inflammations of these parts which occur in the Exanthemata, or rather, which, as far as relates to practical treatment, seem to constitute the essential part of the disease, the mere exanthema being for the most part of subordinate importance. In the milder cases little is to be apprehended: but when the inflammation of a part of the mucous membrane is severe, it is more apt to run a rapid course when attendant on the exanthemata, than under other circumstances. In these, as in the former class, each part may become the principal seat of the disease. In measles, small-pox, and scarlet fever, I have not met with any severe case, in which the mucous membranes were not implicated, and the proof, in many cases, has been rendered evident on dissection. Neither have I met with any case which terminated fatally, where, on careful examination, these membranes were found to be free from disease.

"If HOOPING-COUGH may be considered as a specific disease, it may be classed with the above; for, whoever will investigate it by pathological anatomy, will find *that it is essentially an inflammatory disease of the mucous membrane, affecting the larynx, trachea, and bronchia:* although in advanced stages the patients often die from the affection of the brain which supervenes. The opinions respecting *spasm* in this disease have done much to mislead the profession, and when, like many others, I acted upon the received opinions of the supposed spasmodic nature of hooping cough, I had often to regret the insufficiency of such medical treatment. I therefore determined to divest myself as much as possible of preconceived opinions, and to investigate the nature of the disease through the medium of pathological anatomy.

"I soon found that the usual mode of making the examination of morbid appearances, that is, examining only the head, the chest, and the abdomen, afforded no clue to the nature of the disease; and, though the lungs were generally more or less affected, there did not appear sufficient organic derangement to have produced death. I then determined to examine carefully the whole of the air passages, and there found sufficient to account for the death; for it became evident that it must have arisen from suffocation. The larynx, in every instance, afforded indications of increased vascularity, and the accumulation of mucus, or puriform fluid in the air passages, was sufficient to prevent respiration. These circumstances, collated with the symptoms during life, rendered it easy to conceive that the whole might be the result of inflammation; and the fortunate result of a mode of treatment founded on this principle, (as well as the repeated observation of the morbid appearances) have confirmed me in this opinion. Indeed, I may add, that it was the detection of this remarkable connection between these diseases and inflammations of particular parts of the mucous membranes which led me to more extended investigation." 118.

Mr. A. thinks that, by patient investigation, in any given case, the seat and nature of the disease will be clearly recognized—its degree of intensity ascertained—and an efficient plan of treatment indicated.

"It must be obvious to every one, who has at all minutely observed the phenomena of disease, that irritation or inflammation, either of the skin, or of the mucous membranes, produces an increased secretion or exudation from the affected part.—The former may be observed on the application of a blister, in scalds, erysipelas, &c.; the latter in cases of common catarrh, ophthalmia, gonorrhœa, dysentery, &c.

"We may observe in mild cases of the latter class, take catarrh for instance, that the first indication of local disease is uneasiness combined with some degree of swelling or distention of the affected parts, that this is followed by a thin liquid secretion from these parts; that the secretion becomes more tenacious, and, if the excitement have been sufficiently intense, is succeeded by a puriform discharge. As we observe that this progress takes place in a greater or less degree when the disease is left to its natural course, are we not justified in inferring, that the increased secretion is the means which nature employs for the removal of the disease, as dropsy appears to be one of the modes in which increased vascular fulness spontaneously terminates?

"Although the increased secretion from the mucous membrane lining the organs of respiration, is one of the modes by which recovery is effected, yet its excess, similar to what we witness in many dropsical diseases, is attended with great danger. No sooner does the secretion into the air-passages exceed that which can be removed by exhalation and expectoration, than accumulation begins, and a part of the cells of the lungs, which should receive air, becomes filled with the secreted fluid ; this increasing, must necessarily prevent the due performance of the function of respiration—that function without which life cannot long exist,—the blood no longer is changed from the dark or venous, to the vermillion hue, and the colour of the body partakes of the leaden or livid shade ;—if the accumulation proceed, respiration becomes more and more obstructed, the phlegm, may be heard rattling in the air-passages,—and the patient sinks exhausted and suffocated." 120.

In measles we trace the first appearances of inflammation in the watery and suffused eyes, the discharges from the nostrils—the sneezing, and other catarrhal symptoms, evincing phlogosis of the mucous surfaces lining these parts, while inspection shews its actual existence in the visible parts of the throat.

"Examinations of morbid appearances, after death, in cases of measles, have shewn the lining membrane of the air passages in a highly vascular and inflamed state ; the air passages themselves filled in so large a pròportion with mucus, or puriform fluid as to prevent the oxydation of the blood, and consequently, to destroy life. In cases where the disease has been more protracted, the air passages have not been filled with mucus, but chiefly with pus, sometimes mixed with air and mucus, extending throughout the most minute ramifications of the bronchia, and rendering a great part of the lungs much more dense than in the natural state.

"The lungs themselves have less frequently exhibited signs of inflammation ; at least, those recent adhesions of the pleura, so commonly met with, have rarely appeared. So decidedly has this been the case, that I have known several instances where the examination of the head, the chest, and the abdomen, have afforded no satisfactory explanation of the cause of death, until the larynx, trachea, and bronchia were examined, when the cause of death became apparent. In some cases, sloughing of the tongue or fauces has intervened ; and I have known more than one instance where ulceration of the larynx had taken place. In one case, a considerable part of the epiglottis was destroyed by ulceration, and as might be expected, every attempt to swallow, particularly liquids, produced great distress, and violent cough, almost bordering on suffocation." 122.

The greater number of the tracheal and bronchial affections in the advanced stages of measles assume the acute form, and require very active treatment. Mr. A. has seldom found the mucous membrane of the alimentary canal with the exception of the pharynx, affected in measles. The brain is less frequently affected than in hooping cough.

From these observations the principal indications of treatment will hinge on the moderation of general febrile symptoms, and the removal of the inflammation of that particular part of the mucous surfaces which is the chief seat of the disease. Mr. Alcock candidly observes, that the means of treatment must be left to the discretion of the individual practitioner, the grand object being to subdue the disease by the mildest means consistent with the welfare and safety of the patient.

### SMALL POX.

"It is sometimes of use to trace the steps by which any useful discovery has been made, or any practical fact has been ascertained, as it will generally be found, that whatever has been useful, has been the result of induction from the accurate observation of obvious, and frequently of well-known facts.

"Several years ago, and previous to my residence in London, my attention had been directed to the puriform discharge from the internal ear, from having observed a case or two which had terminated fatally, with well-marked symptoms of affection of the brain. On tracing a number of cases, I found that they had generally originated in, or followed some acute disease, such as measles, small-pox, or scarlet fever. This circumstance led me to examine the internal ear, when inspecting morbid appearances after death, and I soon found, that in almost every fatal case of the above named diseases, there was more or less of effused fluid in the cavity of the tympanum, which varied from the appearance of a thin and bloody mucus, to that of well-formed pus; the membrane of the tympanum remaining entire.

"It scarcely appears necessary to notice, that in the year 1812, on presenting a paper, *On the purulent discharge from the Ear*, to the Westminster Medical Society, I illustrated this fact by a recent preparation of the temporal bone, removed from a patient who had died of confluent small-pox, shewing the cavity of the tympanum filled with pus As might be expected, I invariably found this state had been preceded by considerable mischief in the throat, in many instances even to the throwing out of coagulable lymph on the surface. The similarity of this adventitious production to the membrane of croup, and the invariable affection of the larynx and pharynx, as exhibited by dissection, soon convinced me that the immediate cause of death was suffocation, and the further examination of the air passages, confirmed me in this conviction. This fact of similarity of diseased action in parts so essential to life, required no great ingenuity to be reduced to practical use. Notwithstanding the prevailing opinion that confluent small-pox was a disease of debility, of putrescency, &c. yet if the inflammation of the internal organs produced death in croup, and the morbid appearances in confluent small-pox bore a strong resemblance to the former, I could not see why the treatment of small-pox, as a decided inflammation, should not be as efficacious as that of croup; in which, decisive antiphlogistic measures, if used sufficiently early, seldom fail to remove the disease. I determined to put it to the test, and the result exceeded my warmest expectation; and some of my professional friends have since used the same plan with equally happy success. Perhaps some may consider the fact as of little importance; I can only state my belief that in very many instances it has been the means of saving the lives of individuals who have been under my care, and who, I am persuaded, would not have recovered under the usual mode of treatment." 125.

Mr. Alcock appears to think, and we agree with him, that what have been termed pustules on the mucous membranes, in small-pox, ought to be termed aphthous ulcers. After detailing the dissection of a case of confluent small-pox which terminated fatally, and where the trachea and bronchia were filled with greyish puriform matters, while the larynx and pharynx were swollen and covered with coagulable lymph, the œsophagus, stomach, and intestines being pale and free from disease, Mr. A. concludes thus:—

"The treatment of small-pox upon the principles above described, forms so good an illustration of the value of those principles when reduced to practice, that I cannot refrain from recapitulating, as a general fact, that the danger of small-pox has been in the ratio of the laryngeal, tracheal, and bronchial inflammation, and that this inflammation has usually borne a strict relation to the aphthous eruption, (or what would have been pustular eruptions if situated on the skin,) which are to be found in the throat. A reference therefore to the state of the throat in the earliest days of the eruption, has formed an almost unerring guide to the future practice, and in nearly all cases of confluent small-pox, when seen thus early, a decidedly depletory and antiphlogistic system of practice, has conquered the disease, and saved the person's life. So certainly has this treatment answered the intended purpose, that in many cases the disease has been cut short, the eruptions have run through their course more rapidly than under ordinary circumstances, the secondary fever, as it has been called, has not occurred, and the person's countenance has been left free from pits, or the marks have been comparatively slight." 129.

### SCARLET FEVER.

Mr. Alcock thinks that the mortality in this disease has been considerably over-rated—although, in this opinion he is aware that he differs from authors of great talents and experience. But the fact is, that we may live a long time and meet with few bad epidemics of scarlet fever; but when the malignant cases do occur, they defy the best and most energetic modes of treatment.

### HOOPING-COUGH.

"The symptoms attending this severe and often fatal disease are known to almost every one, so that detailed description of them is unnecessary. The great severity of the paroxysms when the disease is fully formed, and the immediate relief which follows the expulsion of the tenacious matter which is expectorated, or often swallowed by young children, are generally sufficient to distinguish this disease. The early symptoms are so perfectly similar to those of common catarrh, that I know of no diagnostic by which they may be discriminated. Catarrh is as obviously an inflammation of a mucous membrane as is gonorrhœa;—what then should induce us to consider hooping-cough as a spasmodic disease, because the muscles of respiration are occasionally called into violent action. As well might we designate the violent efforts which instantly occur on any extraneous body entering the glottis a spasmodic disease; and with equal wisdom to the spasmodic treatment of hooping-cough, set about treating the spasm, regardless of the cause which produced it, and leave the morsel in the larynx till the patient was suffocated; and then console ourselves with the reflection, that although the patient had died, the friends had the satisfaction of knowing *that every thing had been done which could be done;* and thus go on with the next and the next. I have repeatedly ascertained, by dissections of patients who have died of hooping-cough, that the larynx invariably exhibited signs of inflammation often to so great an extent as by its swelling to close mechanically the glottis;—often the exudation of coagulated lymph was found near the larynx; the mucous or lining membrane of the trachea and bronchia was much increased in vascularity, and the cavities of the latter were filled with fluid more or less mixed with air,—the appearance of the fluid varying from that of thin mucus to perfectly formed pus.

"With these facts as a basis, is it unreasonable to suppose that the inflammation of the mucous membrane lining the various parts of the organs of respiration, should produce the tenacious mucus, which is from time to time expectorated? that the accumulation of this matter impedes respiration, and acts as an extraneous body upon the larynx; that the cough is a mere natural effort to expel the offending matter, and its violence is in direct ratio with the tenacity of the phlegm secreted;—as we often find that spontaneous vomiting frequently terminates the paroxysm, by bringing away the secretion adhering about the top of the larynx, and which the cough had not been sufficient to dislodge? This irritating cause being removed, there is soon a cessation of all the urgent symptoms until its accumulation, or other accidental cause, produce the recurrence of paroxysm. If the disease be not subdued, either by natural means or by appropriate remedial treatment, the excessive secretion continues, accumulation takes place in the bronchia and in the air cells of the lungs, and consequently the blood is prevented from that intimate contact with the air respired, which is so essential to life, that the continuance of this state terminates in suffocation.

"The treatment must not be confined to the mere alleviation of symptoms, but must, to be successful, strike at the cause. When the inflammatory affection is such as to call for further measures than those of regimen, temperature, &c. depletion, active depletion, must be used ;—and frequently the combination of blood-letting, both general and local, will afford more relief than either of them separately. These measures will be greatly assisted by such remedies as tend to restore the equilibrium of the circulation—the distribution of blood generally being in excess in the internal organs, and deficient in the skin and contiguous parts. The occasional exhibition of emetics is also of use, and still more particularly such medicaments as produce a copious mucous secretion from any large surface of the mucous membranes, as those of the stomach and intestines." 133.

We have thus given an analysis of the only part of Mr. Alcock's work which is susceptible of analysis. Our readers will judge from this specimen of the general value and tenor of the volume under consideration. We have no doubt that these lectures had a better effect during oral delivery, than they will have on perusal. The language is often diffuse and colloquial, far better suited for the theatre than the closet. Mr. Abernethy's lectures afforded an excellent illustration of this remark. None were more popular in the class-room—none more disappointing when committed to the press.

